# Raspberry Powder (Rubus idaeus L.) as a Natural Preservative in Aerobically Packaged Ground Beef: The Phytochemical Profile and Effect on Lipid Oxidation, Color Deterioration, and Microbial Growth during Storage

**DOI:** 10.1021/acsomega.5c02471

**Published:** 2025-05-16

**Authors:** Muhammet İrfan Aksu, Emre Turan, Aysel Gülbandılar, Nevzat Konar, Faruk Tamtürk

**Affiliations:** † Atatürk University, Faculty of Agriculture, Department of Food Engineering, 25100 Erzurum, Türkiye; ‡ 187474 Ordu University, Faculty of Agriculture, Department of Food Engineering, 52200 Ordu, Türkiye; § Ordu University, Institute of Science, Department of Food Engineering, 52200 Ordu, Türkiye; ∥ 53004 Eskişehir Osmangazi University, Faculty of Agriculture, Department of Food Engineering, 26160 Eskişehir, Türkiye; ⊥ 37504 Ankara University, Faculty of Agriculture, Department of Dairy Technology, 06170 Ankara, Türkiye; # DÖHLER Food and Beverage Ingredients R&D Center, 70100 Karaman, Türkiye

## Abstract

In this study, the effect of encapsulated raspberry powder (EnRP) as a natural antimicrobial and antioxidant on the quality characteristics and shelf life of ground beef packaged under aerobic storage conditions was investigated. For this purpose, the bioactive compound profile, antioxidant, and antimicrobial properties of EnRP were determined, and ground beef samples treated with EnRP (0.0, 1.0, 2.0, and 3.0%) were aerobically packed and stored at 2 ± 0.5 °C for 5 days. Increasing EnRP level decreased (*P* < 0.01) the pH values of samples, and the lowest values recorded in 3% EnRP treatment (*P* < 0.05). Lipid oxidation and color values of ground beef were also affected by the addition of EnRP (*P* < 0.01) and the groups containing 2 and 3% EnRP had the highest a* (redness) and lowest TBARS values (*P* < 0.05). Lipid oxidation at the end of storage was inhibited by up to 81.24% compared to the control, depending on the EnRP level (*P* < 0.05). The addition of EnRP also had significant effects on the microbiological quality of meat samples (*P* < 0.01) and lower counts were found in the samples containing EnRP compared to the control (*P* < 0.05). These results showed that EnRP can be used to preserve the quality characteristics of ground beef stored under aerobic packaging conditions.

## Graphical abstract

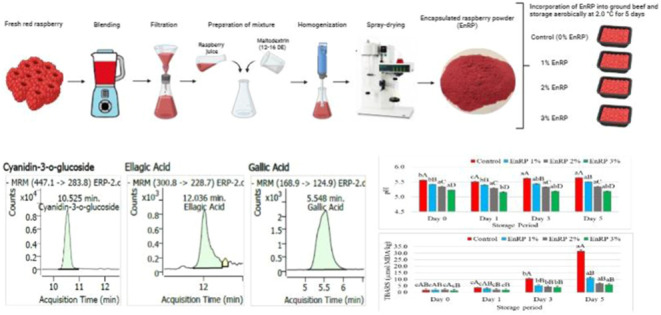

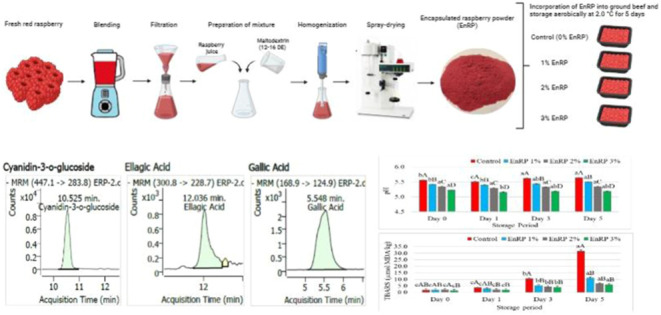

## 1 Introduction

Fresh beef is processed in different ways and offered for consumption in forms such as minced meat and cubed and sliced meat. The most common among these is ground beef. Due to the expansion of the surface area of meat during grounding, these meats become susceptible to oxidation. During this process, due to the deterioration of the muscle membrane, the fat membranes are more exposed to oxygen, metal ions, and temperature, and oxidation is accelerated.^1^ Expansion of the meat surface area not only affects lipid oxidation but also causes the oxidation of color pigments and proteins. Oxidation products not only reduce product quality but also increase health risks by producing toxic substances (malondialdehyde, 4-hydroxynonenal, cholesterol oxides, etc.).^2–4^ For this reason, the use of antioxidants in ground beef and similar meat products is important. However, interest in synthetic antioxidants, which have the potential for widespread use, is gradually decreasing due to their potential harmful effects on health.^2^ In this context, the use of natural antioxidants comes to the fore. Recent studies have investigated the use of powders and extracts from natural sources such as Ataulfo mango peel,^4^ raspberry,^5^ grape (Vitis vinifera) and orange (Citrus reticulata) byproducts,^6^ passion fruit (Passiflora edulis) residues,^7^ black carrot (Daucus carota L.),^8,9^ and prickly pear^10^ as natural antioxidants in meat and meat products.

Ground beef is a product that is easily spoiled in terms of its microbial properties. It is a good environment for microorganism growth in terms of nutritional components, oxygen, pH, and storage temperature and conditions. *Pseudomonas, Carnobacterium*, *Clostridium*, B. thermosphacta, psychrotrophic lactic acid bacteria, and *Enterobacteriaceae* species are bacteria that cause microbial spoilage in raw meat stored in cold temperatures.^11^ As fresh meat is among the foods most susceptible to spoilage, combined treatments are often used to preserve meat. The short storage life of fresh beef (ground beef, etc.,) of 1–2 days at refrigerator temperature without any processing can be extended by packaging or by adding various antimicrobial and antioxidant substances.^1,10^ Compared with vacuum packaging, microbial spoilage is faster in aerobically packaged ground beef. In this context, the prevention of microbial spoilage in aerobically packaged ground beef is of great importance. For this reason, adding antimicrobial substances to the product is important in terms of preserving the quality and extending the shelf life. However, according to Regulation EU 601,^12^ although the addition of antioxidants to such products is permitted, the use of antimicrobials is not permitted.^10,12^ Therefore, microbial spoilage can be prevented by adding natural antimicrobial substances to these products.

Raspberries are valued for their taste, color, and health benefits due to bioactive compounds such as anthocyanins, ellagitannins, and vitamins.^13^ Many studies have reported various bioactive properties of raspberry, including antioxidant and antimicrobial activity.^14–16^ However, their high perishability requires rapid processing after harvesting, e.g., by drying.^17^ In this context, convective drying is cost-effective but impairs the stability of the bioactive components. Lyophilization preserves phytonutrients better, but its disadvantage is that it is an expensive method. Thus, spray-drying using a carrier agent is a more economical method that produces stable, nonsticky fruit powders while preserving bioactive components for industrial use.^18–20^

Despite its unique red color and strong bioactive properties, there are limited studies on the use of raspberry in meat products such as burgers,^21,22^ beef patties,^23^ pastırma,^24^ vacuum-packed ground beef,^5^ cemen paste,^25^ and chicken nuggets.^26,27^ To the best of our knowledge, the influence of encapsulated raspberry powder on the quality of aerobically packaged meat has not yet been investigated. Accordingly, the objectives of this study were (I) to investigate the bioactive compound profile and antioxidant and antimicrobial properties of EnRP, and (II) to determine the changes in color, microbial quality, and oxidative stability of EnRP-treated ground beef during storage at 2 ± 0.5 °C for 5 days under aerobic conditions.

## 2 Material and Methods

### 2.1 Preparation of Encapsulated Raspberry Powder (EnRP)

EnRP production was carried out according to Gagneten et al.^18^ and Aksu et al.^5^ For this purpose, fresh raspberry fruits (Rubus idaeus L. cv. Heritage) supplied from local farmers/greengrocers operating in Karaman, Türkiye were used as raw materials. In this study, in addition to the drying and/or encapsulation conditions performed using the spray-drying technique with different raspberry products,^18,19,28^ a wall material commonly used for anthocyanin encapsulation was selected.^29^ In production, raspberry juice (dry matter: approximately 30.0%) was used by mixing with maltodextrin (DE 12–16) at a 60:40 ratio. EnRP production was carried out using a laboratory-scale spray dryer (Buchi B290, Switzerland), with the following parameters: inlet temperature: 160 °C, outlet temperature: 90 °C, feed rate: 8 mL/min, and air flow rate: 600 L/h.

### 2.2 Characterization of EnRP

#### 2.2.1 Moisture Content, pH, and Instrumental Color Values of EnRP

The moisture content was determined by following the procedure of heating the sample in an oven at 70 °C for 6 h, until a constant weight was reached. For constant weight control, the weight was monitored regularly until the difference between two consecutive measurements was less than 0.01 g. A calibrated benchtop pH meter (Mettler Toledo S210, Switzerland) was used for the pH measurement of the EnRP-distilled water mixture (1:10 w/v). Lightness (L*), redness (a*), and yellowness (b*) color values of EnRP were determined using a Minolta (CR-410, Osaka, Japan) colorimeter calibrated with a white plate. The measuring area, illuminant, and standard observer for the instrument were Φ50, D65, and 2°, respectively.^30^

#### 2.2.2 Bioactive Compound Content and Antioxidant Capacity of EnRP

Extraction of phenolic compounds in EnRP was carried out using an acidified methanol-distilled water mixture (70:30 v/v) containing 0.1% HCl. The obtained extract (0.5 mg/mL) was filtered through a syringe filter (0.45 μm pore size, Isolab, Germany) and used in the phenolic compound profile and antioxidant capacity analyses. Total phenolic content (TPC) based on the Folin-Ciocalteu test was determined as gallic acid equivalents (GAE)/100 g dry weight (dw).^31^ Total flavonoid content (TFC) was calculated as mg quercetin equivalents QE/100 g dry weight (dw) according to the colorimetric aluminum chloride test.^32^ The total anthocyanin content (TAC) of EnRP was determined by spectrophotometric (Jasco, V-730) monitoring of the pH-dependent color change resulting from treatment of the extract with 0.025 M potassium chloride (pH 1.0) and 0.4 M sodium acetate (pH 4.5) buffer solutions. TAC was expressed as mg cyanidin-3-glucoside (C3G)/100 g dw.^33^ The vitamin C content of EnRP was determined by following the 2,6-dichlorophenol indophenol titrimetric method. The *in vitro* antioxidant activity of EnRP was evaluated using the cupric ion reducing antioxidant capacity (CUPRAC), 2,2′-Azino-bis­(3-ethylbenzothiazoline-6-sulfonic acid) diammonium salt (ABTS) antiradical activity, and Ferric reducing antioxidant power (FRAP) assays developed by Apak et al.,^34^ Re et al.,^35^ and Benzie and Strain,^36^ respectively. The results of all three antioxidant activity tests are presented as μmol of trolox equivalent (TE)/mg of dw.

#### 2.2.3 Phytochemical Profile of EnRP

Phytochemicals in EnRP were quantitatively determined using UHPLC-ESI-MS/MS according to the method reported by Can et al.,^37^ employing an Agilent 1290 Infinity UHPLC system (Agilent Technologies, Palo Alto) connected via an electrospray ionization source (ESI) to an Agilent 6460 Triple Quadrupole MS-MS (Palo Alto, CA,). The separation was carried out using a C18 reversed phase analytical column (Zorbax SB-C18) (4.6 × 100 mm, 3.5 μm) whose temperature was held constant at 30 °C. The elution gradient consisted of mobile phase A (water and 0.1% formic acid) and mobile phase B (acetonitrile and 0.1% formic acid). The gradient was applied to mobile phase B at varying concentrations (5 to 5–20–90–90–5–5) after 0–4–7–14–15–15.1–20 min, and the flow rate of mobile phase B was 0.4 mL/min. The operating conditions were as follows: drying gas (nitrogen) temperature, 350 °C; drying gas (nitrogen) flow, 12 L/min; nebulizing gas (nitrogen) pressure, 55 psi; capillary voltage, 3.5 kV; sheath gas heater, 250 °C; and sheath gas flow, 5 L/min. Mass spectrometry analysis was performed in the multiple reaction monitoring (MRM) mode by screening specific precursor phytochemical-to-fragment ion transitions. Agilent MassHunter Workstation (Agilent) software was used for data collection and analysis.

#### 2.2.4 Antimicrobial Potential of EnRP

The microtube dilution technique was applied to determine the minimum inhibitory concentration (MIC) of EnRP that inhibited the growth of the tested microorganisms.^38^ In the determination of antimicrobial activity, the bacterial strains consisted of four Gram-positive (Staphylococcus aureus, Bacillus subtilis, Enterococcus faecalis, and Listeria monocytogenes) and two Gram-negative (Pseudomonas aeruginosa and Escherichia coli). Candida albicans was used to determine the antifungal activity of EnRP. Each microorganism culture was incubated in Muller Hinton Broth at 37 °C overnight and adjusted to a concentration of approximately 10^8^ cfu/ml (with 0.5 McFarland) in tubes containing 15 mL of double-strength Muller Hinton Broth. For the antimicrobial extract, 20 mg of EnRP was dissolved in 10 mL of dimethyl sulfoxide (DMSO) solution, and the same procedure was followed for the standard antibacterial (Levofloxacin, Cefepime, and Vancomycin) and the antifungal (Flucanozole) compounds. Dilutions of EnRP and control antimicrobial compounds were prepared with 1000 μL of DMSO solution and 1000 μL of sterile distilled water in a 1:1 ratio. The MIC test was performed in three independent experiments in sterile 96-well microplates. The first 11 wells of the microplate contained 2-fold serial dilutions (from 2000 to 1.95 μg/mL) of the antimicrobial extracts, while the 12th well contained only MHB for sterility control. The prepared plates were incubated for 24 h at 37 °C. The lowest dilution ratio at which turbidity was observed in the plate wells was determined as the MIC value.

### 2.3 Ground Beef Production, Packaging, Storage, and Analysis

The meat used in this study for the preparation of ground beef was procured from a private food enterprise (Namsan Inc.) in Eskişehir/Türkiye. Muscles for ground beef were obtained from the round region of carcasses of 18–22-month-old male cattle (Brown Swiss crossbred). Meat taken from beef carcasses was brought to the laboratory of Eskişehir Osmangazi University, Department of Food Engineering, under hygienic conditions and in a cold chain, and was used as a research material after being passed through a meat grinder with a mirror hole diameter of 3–5 mm. Then, ground beef was stored at 4 °C until used (approximately 1 h), i.e., until raspberry powder was added and packaged. Three independent meats were used in the preparation of ground beef; that is, independent batches of different meats were prepared for each repetition. The ground beef prepared for each repetition was first divided into 4 treatment groups (4 for each batch, 12 in total) for EnRP addition (1.0, 2.0, and 3.0%) and control (0.0%) treatment. Then, the ground beef prepared for each treatment group (control and EnRP added) was divided into 4 allocations of 200 g considering the storage periods (0, 1, 3, and 5 days). Thus, a total of 48 (four treatments x four storage periods x three batches) ground beef samples were prepared for three independent repetitions. All of the prepared samples were placed in polystyrene plates and packaged with a single-layer stretch film. Ground beef samples packaged in this way were stored at 2 ± 0.5 °C for 5 days and analyzed on the zeroth, first, third, and fifth days of storage. In the ground beef samples stored, the pH, TBARS, color (L*, a*, b*, C*, and *h°*) analyses, aerobic psychrotrophic bacteria, aerobic mesophilic bacteria, *Micrococcus/Staphylococcus*, Staphylococcus aureus, *Pseudomonas* and *Enterobacteriaceae* counts were performed on each storage day.

#### 2.3.1 Analysis of Ground Beef

##### 2.3.1.1 pH Value

The pH values of the ground beef samples were determined by using a pH meter. The pH meter is calibrated with buffer solutions (pH 4, 7, and 10) before use. The 10 g of the beef meat samples were weighed in three parallel, and 100 mL of pure water was added to them. The mixture was homogenized with Ultra-turrax (IKA T25, Staufen, Germany), and the pH value was determined.

##### 2.3.1.2 Instrumental Color Values

The color values (L*, a*, and b*) of ground beef were measured using a Minolta (CR-400, Minolta Co, Osaka, Japan) colorimeter device on a white background (illuminant D65, 11 mm measuring aperture, observer angle 2°). Then, from the a* and b* values, chroma (C*) and hue angle (h°) values were calculated using these formulas: Chroma (C*) = [*a* ^2^ + *b* ^2^]^1/2^ and hue angle (h°) = tan^–1^[*b*/*a*], which express the intensity and redness discoloration, respectively. Color intensities were measured according to the criteria given by the International Illumination Commission CIELAB (Commision Internationele de I’e Clairage). According to these criteria, L* value; L* = 0, black; L* = 100, white (darkness/lightness); a* value; +*a* = red, −a* = green and b* value; +b* = yellow, −b* = blue indicate color intensities.

##### 2.3.1.3 Thiobarbituric Acid Reactive Substance Values

Lipid oxidation in ground beef samples was performed by determining the thiobarbituric acid reactive substances (TBARS value) using the method modified by Aksu and Turan.^8^ The method was applied as modified in order to eliminate the pinking effect of raspberry powder independent of malondialdehyde (MDA) formation.^8^ The TBARS values were calculated as μmol of MDA/kg of meat.

##### 2.3.1.4 Total Aerobic Mesophilic and Total Aerobic Psychrotrophic Counts

Plate Count Agar (PCA, Oxoid) medium was used to determine the total mesophilic and total psychrotrophic bacterial counts in ground beef. Inoculation was performed by spreading appropriate dilutions, and after inoculation, the Petri plates were incubated at 10 °C for 7 days for the psychrotrophic bacteria count and at 30 °C for 48 h for the total mesophilic bacteria count.

##### 2.3.1.5 *Micrococcus/Staphylococcus* Count

For the *Micrococcus/Staphylococcus* enumeration of ground beef samples, Mannitol Salt Phenol Red Agar (Merck) was used. Appropriate dilutions were inoculated on Mannitol Salt Agar plates by the spreading method, and the plates were incubated aerobically at 30 °C for 48 h. The count was determined by taking into account catalase-positive cocci.

##### 2.3.1.6 Staphylococcus aureus Count

Baird Parker Agar medium with a 5% egg yolk tellurite emulsion was used to enumerate Staphylococcus aureus from ground beef samples. The plates, which were inoculated according to the surface spreading method from appropriate dilutions, were incubated at 37 °C for 30–48 h. Gray and black colonies with a clear zone around them as a result of incubation were evaluated as suspicious staphylococcal colonies. Later, a coagulase test was applied to suspected staphylococcal colonies, and coagulase-positive colonies were defined as the staphylococcus genus.

##### 2.3.1.7 *Pseudomonas* Count

CFC agar (*Pseudomonas* Agar Base-Oxoid CM 0559) was added to the CFC supplement (Oxoid SR 0103) according to the procedure used to determine the counts of *Pseudomonas* in ground beef samples. Planted according to the surface spreading method, and Petri dishes were incubated under aerobic conditions at 25 °C for 48 h. The oxidase test was performed on the colonies that grew as a result of incubation, and the counts were determined by counting the oxidase-positive colonies.

##### 2.3.1.8 *Enterobacteriaceae* Count

Violet Red Bile Dextrose Agar (VRBD Agar; Merck) was used to observe the counts of *Enterobacteriaceae*. Petri plates were incubated at 30 °C for 48 h under anaerobic conditions (Anaerocult A, Merck). After incubation, the counts of *Enterobacteriaceae* were determined by counting red colonies larger than 1 mm.

### 2.4 Statistical Analysis

The research was conducted with a randomized plot factorial design by applying four different EnRP levels (0.0%, 1.0%, 2.0%, and 3.0%) in four different storage periods (0, 1, 3, and 5 days) with three independent replications (batch). All experimental data from the aerobically packed ground beef samples were subjected to analysis of variance using a general linear model (GLM), with EnRP levels and storage period as fixed factors and batch as a random factor. In cases of significant differences between the means, Duncan’s multiple range tests (*P* < 0.05) were applied for comparison. All statistical analysis was performed with the SPSS 25.0 package program (BM SPSS Inc.). The mean ± standard error was used in tables and figures for the presentation of results.

## 3 Results and Discussion

### 3.1 Physicochemical Properties, Bioactive Compounds, and Antioxidant Capacity of EnRP

Physicochemical and antioxidant capacity properties of EnRP are presented in Table 1. The moisture content and pH of EnRP were 4.77% and 3.38, respectively, similar to values reported in the literature for red raspberry powders.^8,19,26^ Regarding the instrumental color values, L*, a*, and b* color values of EnRP were 38.75, 34.14, and 8.31, respectively. Our instrumental color values for spray-dried raspberry powder were higher than those reported by Yu et al.^17^ but were lower than the values found by Aksu et al.^5,25^ This may be attributed to differences in factors such as measuring device, fruit genotype and maturity, drying method, and drying conditions (carrier type, carrier:fruit ratio, temperature, etc.).

**Table 1:** Physicochemical Properties, Antioxidant Capacity, and the Phenolic Profile of Encapsulated Raspberry Powders (EnRP)

parameter	mean ± SE
total phenolic content (mg GAE/100 g)	558.23 ± 6.08
total flavonoid content (mg QE/100 g)	291.6 ± 5.48
total anthocyanin content (mg cyanidine 3-glucoside/100 g)	121.34 ± 5.87
copper-reducing antioxidant capacity (CUPRAC) (μmol TE/g)	103.13 ± 17.03
ABTS radical scavering activity (ABTS-RSA) (μmol TE/g)	52.30 ± 5.41
ferric reducing antioxidant power (FRAP) (μmol TE/g)	698.0 ± 16.06
vit C content (ppm)	259.4 ± 6.99
moisture content (%)	4.77 ± 0.01
pH	3.38 ± 0.02
L*	38.75 ± 0.44
a*	34.14 ± 0.66
b*	8.31 ± 0.18

phenolic profile			
compounds	category	class	content (ppm)
quinic acid	organic acid		1927.11 ± 1.73
fumaric acid	organic acid		9.33 ± 0.49
gallic acid	phenolic acid	hydroxybenzoic acids	63.81 ± 2.59
4-OH-benzoic acid (4-hydroxybenzoic acid)	phenolic acid	hydroxybenzoic acids	4.46 ± 0.01
vanillic acid	phenolic acid	hydroxybenzoic acids	0.27 ± 0.19
syringic acid	phenolic acid	hydroxybenzoic acids	0.10 ± 0.14
p-coumaric acid	phenolic acid	hydroxycinnamic acids	2.55 ± 0.11
sinapic acid	phenolic acid	hydroxycinnamic acids	4.89 ± 0.19
ferulic acid	phenolic acid	hydroxycinnamic acids	7.14 ± 0.7
rosmarinic acid	phenolic acid	hydroxycinnamic acids	0.63 ± 0.01
cyanidin-3-rutinoside	flavonoid	anthocyanins	11.9 ± 0.59
cyanidin-3-o-glucoside	flavonoid	anthocyanins	476.12 ± 0.21
peonidin-3-o-glucoside	flavonoid	anthocyanins	4.39 ± 0.1
catechin	flavonoid	flavanols	1.07 ± 0.04
epicatechin	flavonoid	flavanols	19.34 ± 0.25
naringin	flavonoid	flavanones	0.06 ± 0.08
quercetin	flavonoid	flavonols	14.08 ± 1.15
ellagic acid	hydrolyzable tannins	ellagitannin	180.21 ± 14.96
vanillin	phenolic aldehyde		1.84 ± 0.04
resveratrol	stilbenes		0.11 ± 0.04

The raspberries are good source of phenolic compounds, including flavonoids and anthocyanins.^39,40^ The bioactive compound content and antioxidant activity of raspberry vary over a wide range depending on genotype, harvest season, extraction procedure, storage, processing method, and conditions.^19,41–43^ EnRP had a total flavonoid content (TFC) of 291.6 mg of QE/100 g of dw. Ceylan et al.^15^ determined the average TFC of raspberry fruits as 1035.3 mg QE/100 g dw, while Si et al.^44^ reported the TFC of raspberry powders dried by different methods in the range of 26–30 mg catechin equivalent/100 g dw. Total phenolic (TPC) and total anthocyanin (TAC) contents of EnRP were 558.23 mg of GAE/100 g of dw and 121.34 mg of C3*G*/100 g of dw, respectively. In the literature, similar,^26^ higher^17,19^ or lower^18^ TPC and TAC contents were reported for spray-dried raspberry powders compared to the results of the present study. Vit C is heat-labile and its temperature-dependent loss during spray-drying of raspberries has been reported.^19^ Nevertheless, the vitamin C content of EnRP was competitive with the literature data reported for raspberries.^13,43^

The bioactive components contribute to the large antioxidant capacity of raspberries, the main representatives of which are anthocyanins, ellagitannins and vitamin C.^13,16^ Anthocyanins have higher antioxidant capacity than ellagitannins because they use multiple radical scavenging pathways.^16^ A strong correlation between TPC, TFC, and TAC of raspberry and its antioxidant capacity has been reported in previous studies.^17,19,41,42^ The antioxidant capacity of phenolic compounds present in raspberries is largely related to their radical scavenging activity; this is due to their highly conjugated system and aromatic structure offering the ability to readily donate electrons and hydrogen atoms.^16^ In the present study, the antioxidant activity of EnRP was evaluated in three different assays as CUPRAC, ABTS-RSA, and FRAP, and the values were 103.13 μmol TE/g, 52.30 μmol TE/g, and 698.0 μmol TE/g, respectively. These findings in the present study are consistent with literature data reported for spray-dried red raspberry powders,^5,25^ but lower than those reported for fresh^19,20^ or freeze-dried^8,15,19,44^ ones. The higher juice proportion in the freeze-dried powders could partly explain this fact; however, the significantly lower bioactive compound content and antioxidant activity values observed in the spray-dried powders, despite their juice concentration, suggest that the high temperatures involved in the spray-drying process may have caused a substantial loss of bioactive compounds.^19,45^

### 3.2 Phytochemical Profile of EnRP

Table 1 presents the phenolic compound profile of EnRP. The phenolic compounds identified in EnRP were categorized as phenolic acids (hydroxybenzoic acids, hydroxycinnamic acids), flavonoids (anthocyanins, flavanols, flavonones, flavonols), ellagitannins, phenolic aldehydes, and stilbenes. In the present study, a total of 18 phenolic compounds were detected in EnRP, with cyanidin-3-o-glucoside (476.12 ppm), ellagic acid (180.21 ppm), and gallic acid (63.81 ppm) being the major compounds. The most intensively studied phenolics in raspberries are anthocyanins and ellagitannins.^16^ Anthocyanins are compounds that give color to fruits and also have strong antioxidant properties. The color of red raspberries comes from the anthocyanins. The major anthocyanins in raspberries are cyanidin-3-sophoroside, cyanidin-3-glucoside, cyanidin-3-glucosylrutinoside, and cyanidin-3-rutinoside.^16,41^ In the present study, cyanidin-3-o-glucoside was the anthocyanin with the highest concentration in EnRP and contributed largely to the red color and high antioxidant activity. Similarly, Chen et al.^41^ reported that cyanidin-3-o-glucoside accounted for about 80% of anthocyanins in dark red colored raspberries. Ellagic acid is one of the predominant phenolic acids in raspberries and together with anthocyanins forms the major part of phenolic compounds.^43^ This phytochemical is known to exhibit various biological properties including antiradical, anticancer, antiviral, and antimicrobial activities.^13,46^ In fact, levels of free ellagic acid in raspberries are generally low, but considerable amounts can be detected together with gallic acid as a result of ellagitannin degradation and acid hydrolysis.^13,47^ Gallic acid is one of the most abundant phenolic acids in EnRP and is characterized by its strong antioxidant properties due to its abilities as an electron donor, radical scavenger, and metal chelator.^48^ Flavonoids such as epicatechin (19.34 ppm) and quercetin (14.08 ppm) support the health benefits of EnRP with potent antioxidant effects. Rosmarinic acid, vanillic acid, resveratrol, syringic acid, and naringin were trace amounts of phenolic compounds in EnRP. The individual phenolic compounds of raspberry extracts has been evaluated in previous studies using different extraction procedures and chromatographic analysis tools.^13,17,41^ In this context, the phenolic profile of EnRP is largely similar to that described by Kodikara et al.^39^ In contrast, Marino et al.^40^ identified sanguiin H6, lambertianin C, and sanguiin H-10 isomers as the major ellagitannins and cyanidin-3-sophoroside, cyanidin-3-glucoside, and cyanidin-3-sambubioside as the most representative compounds among the anthocyanins in lyophilized raspberry powders.

Among the organic acids, quinic acid (1927.11 ppm) is the most abundant compound, followed by fumaric acid (9.33 ppm). These organic acids can contribute significantly to the antioxidant capacity and are involved in various metabolic processes.^37^ Accordingly, the bioactive compound content and antioxidant capacity of EnRP appear to be quite satisfactory, making the product attractive as a natural colorant and antioxidant agent.

### 3.3 Antimicrobial Activity of EnRP

The minimum inhibitory concentration (MIC) (μg/mL) values of EnRP on tested microorganism are shown in Table 2. The MIC values of EnRP against B. subtilis, S. aureus, and P. aeruginosa bacteria were determined to be 31.25 μg/mL. The MIC value of EnRP against E. faecalis, L. monocytogenes, and E. coli bacteria was observed as 62.50 μg/mL. Compared to positive standard agents, the antimicrobial activity of the extracts was higher against B. subtilis and similar in inhibiting E. faecalis and E. coli bacteria. Similarly, the antibacterial activity of the extracts against L. monocytogenes was more effective than Vancomycin and weaker than that of other standard agents. S. aureus and P. aeruginosa bacteria were also found to be similar to or more effective than the positive control agents. EnRP had the same MIC value as the reference compound fluconazole against Candida albicans. The results of our study are compatible with those of previous studies. Similar results were generally obtained in studies with raspberry extracts and powder. Nohynek et al.^46^ stated that raspberry extracts were effective against Bacillus cereus. Velićanski et al.^49^ found that extracts of two raspberry varieties showed strong antibacterial activity against P. aeruginosa and B. cereus. Četojević-Simin et al.^14^ determined that raspberry pulp extracts showed an antimicrobial effect against Gram-positive and Gram-negative bacteria and that this effect did not depend on the strain or species. Similarly, Aksu et al.^5^ and Demirbaş et al.^50^ determined that raspberry extracts have strong antibacterial properties. The antimicrobial activity of EnRP can be attributed to ellagitannins, anthocyanins, and phenolic acids and their mixtures in various chemical forms, possibly through multiple mechanisms and synergies.^14,46,50^ In addition, the presence of organic acids and the acidic pH are factors responsible for the antimicrobial activity of EnRP.^5,14^

**Table 2:** Minimum Inhibitory Concentration (MIC) (μg/mL) of Encapsulated Raspberry Powders (EnRP)^a^

treatment/compound (2 mg/mL)	Gram-positive bacteria				Gram-negative bacteria		fungus
	B. subtilis	E. faecalis *(ATCC 9212)*	S. aureus (NRRL B-767)	L. monocytogenes *(ATCC 7644)*	P. aeruginosa *(ATCC 27853)*	E. coli (ATCC 25922)	C. albicans (F89)
EnRP	31.25	62.50	31.25	62.50	31.25	62.50	62.50
vancomycin	250	62.50	31.25	125	62.50	62.50	NT
levofloxacin	62.50	62.50	31.25	31.25	31.25	62.50	NT
cefepime	62.50	62.50	62.50	31.25	31.25	62.50	NT
fluconazole	NT	NT	NT	NT	NT	NT	62.50

a All values are expressed as mean of three replicates. NT: not treated.

### 3.4 pH Values of Ground Beef Samples

A significant EnRP level x storage time interaction was observed on the pH values of aerobically packaged ground beef samples (Table 3; Figure 1a). As presented in Figure 1a, the increase in the EnRP level incorporated into meat samples resulted in a decrease in initial pH from 5.55 to 5.18 (*P* < 0.05). The pH value of aerobically packaged ground beef increased during storage (*P* < 0.05), and the highest increase in this process was in the control samples without EnRP. During storage, pH did not change in samples with 2% EnRP (*P* > 0.05), a partial decrease was observed in samples with 3% EnRP on the third day of storage and increased again, but this increase remained at the level of 5.18. These results show that EnRP had high acidity and decreased the pH of ground beef. Various studies have shown that the pH value increases during storage; however, depending on the properties and effects of the plant extract or powder added to the ground beef, pH may increase or decrease at the beginning of storage.^4,6^ In parallel with our results, Aksu et al.^5^ also stated that the pH value in control samples of vacuum-packaged ground beef increased during storage (from 5.52 to 5.67). The same study also found that the pH values of the samples with raspberry powder were lower than those of the control ground beef and changed less during storage.

**Table 3:** Main and Interaction Effects of EnRP Treatment and Storage Period on pH, TBARS, Instrumental Color Values, and Microbial Counts of Aerobic-Packaged Ground Beef^a^ ^b^ ^c^ ^d^ ^e^

	pH	TBARS	color values					microbial counts (log CFU/g)					
			L*	a*	b*	chroma (C*)	Hue angle (*h* ^*o*^)	AMB	APB	*Pseudomonas*	*M/S*	S. aureus	*Enterobacteriaceae*
EnRP levels (EnRP)													
control	5.58^a^	11.89^a^	44.0^a^	19.5	13.8^ab^	24.3	34.8^a^	5.21^a^	4.96^a^	4.16^a^	3.94^a^	3.49^a^	3.26^a^
EnRP 1%	5.43^b^	5.24^b^	45.3^a^	19.9	14.1^a^	24.2	35.0^a^	4.55^b^	4.63^b^	3.94^b^	3.68^b^	3.37^ab^	2.78^b^
EnRP 2%	5.32^c^	3.80^c^	44.8^a^	20.2	13.2^ab^	24.0	33.8^b^	4.44^b^	4.24^c^	3.55^c^	3.48^b^	3.15^bc^	2.48^c^
EnRP 3%	5.18^d^	3.29^c^	42.5^b^	20.8	12.8^b^	23.9	32.6^c^	4.40^b^	4.16^c^	3.45^c^	3.64^b^	3.02^c^	2.28^c^
SEM	0.008	0.404	0.503	0.416	0.316	0.479	0.340	0.093	0.062	0.069	0.079	0.096	0.080
*P*	**	**	**	NS	*	NS	**	**	**	**	**	**	**
storage period (days) (SP)													
0	5.38^b^	1.84^c^	44.5	22.4^a^	13.9	26.3^a^	30.8^c^	4.06^c^	4.21^b^	3.46^c^	3.40^c^	3.42^a^	2.31^c^
1	5.33^c^	2.57^c^	44.1	21.1^b^	13.3	24.1^b^	33.5^b^	4.60^b^	4.38^b^	3.57^c^	3.69^b^	3.07^b^	2.27^c^
3	5.39^ab^	5.98^b^	44.4	18.4^c^	13.3	22.9^b^	35.7^a^	4.94^a^	4.62^a^	3.85^b^	4.25^a^	3.68^a^	2.95^b^
5	5.41^a^	13.83^a^	43.6	18.6^c^	13.4	22.9^b^	36.2^a^	5.01^a^	4.77^a^	4.22^a^	3.41^c^	2.86^b^	3.27^a^
SEM	0.008	0.404	0.503	0.416	0.316	0.479	0.340	0.093	0.062	0.069	0.079	0.096	0.080
*P*	**	**	NS	**	NS	**	**	**	**	**	**	**	**
interactions													
EnRP × SP	**	**	NS	**	NS	**	**	**	**	**	*	NS	**

a EnRP: Encapsulated raspberry powders; TBARS: Thiobarbituric acid reactive substances (μmol MDA/kg); L*: Lightness; + a*: Redness; + b*; Yellowness.

b AMB: Aerobic mesophilic bacteria; M/S: *Micrococcus/Staphylococcus*; APB: Aerobic psychrotrophic bacteria.

c Significant differences between treatments are indicated by different small letters (a–d) in the respective column (*P* < .05, Duncan’s test).

d Significant differences between storage periods are indicated by different lowercase letters (a–c) in the respective column (*P* < .05, Duncan’s test).

e * *P* < .05; ** *P* < .01; NS: not significant (*P* > .05).

**Figure 1:**
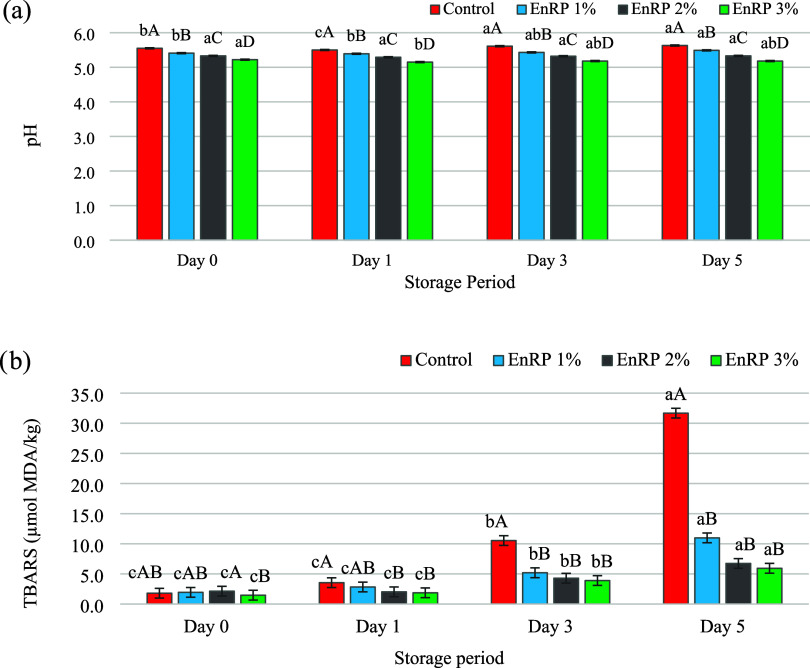
pH (a) and TBARS (b) values of aerobic-packaged ground beef samples treated with different amounts of EnRP during chilled storage. Significant differences between storage periods per EnRP level and between EnRP levels per storage period are indicated by lower and upper case letters in the bar graphs, respectively (*P* < 0.05; Duncan test).

The pH changes in meat depend on intrinsic and extrinsic factors such as initial microbial load, muscle type, packaging method, proximate composition, temperature, oxidative processes, acidity level of additives, and microbial and enzymatic activity.^5,6,8,51,52^ The spoilage during the storage of fresh meat and meat products is generally associated with volatile nitrogenous compounds and the resulting pH change. The increase in pH is caused by the formation of volatile nitrogenous compounds as a result of the effects of various microorganisms and enzymes. The main source of volatile compounds including ammonia and amines, are protein decomposition by microorganisms and enzymes.^6,53^ However, in long-term storage, the degradation of nitrogenous substances due to the excessive count growth of spoilage bacteria contributes to the decrease in pH.^53^ In the present study, the pH of ground beef without EnRP increased during storage due to basic compounds (amines, ammonia, etc.,) produced by microbial growth. As a matter of fact, the initial AMB count (4.40 log cfu/g) in the control group reached 6.09 log cfu/g at the end of day 5 (Table 5) and the change in pH (from 5.55 to 5.63) supported this increase (Figure 1a). However, the pH remained constant during storage in the 2 and 3% EnRP-treated groups (Figure 1a), which can be directly attributed to the acidic character of EnRP (Table 1) and the lower initial microbial load in these groups (Table 5). In ground beef samples containing high concentrations of EnRP, microbial growth was suppressed during storage due to antimicrobial activity from bioactive compounds, and the change in pH values was limited in these groups (Table 5). Consequently, the EnRP contributed to the maintenance of a stable pH in aerobically packaged ground beef.

### 3.5 Lipid Oxidation in Ground Beef Samples

The EnRP level (*P* < 0.01), storage period (*P* < 0.01), and storage period x EnRP level interaction (*P* < 0.01) had significant effects on the TBARS values of aerobically packaged ground beef (Table 3). The values in control samples, which were 1.80 μmol MDA/kg at the beginning of storage, increased to 31.67 μmol MDA/kg (2.28 mg MDA/kg) on the fifth day. This increase could be attributed to the continuous decomposition of hydroperxides due to oxidation during the storage process.^54^ The increase in the TBARS value during storage was also found in previous studies.^5–7^ Incorporation with EnRP decreased the TBARS values of the ground beef samples compared to the control, and this was dependent on the EnRP level (*P* < 0.05; Figure 1b). On day 5 of storage, the TBARS values of the EnRP-containing groups were determined in the range of 5.94–10.98 μmol MDA/kg. In other words, the decrease in TBARS values at the end of storage of aerobically packed ground beef samples with 1, 2, and 3% EnRP compared to the control was 65.33, 78.72, and 81.24%, respectively. In particular, less lipid oxidation occurred in samples with 3% EnRP during storage, and TBARS values were determined to be 1.47 and 5.94 μmol MDA/kg (0.42 mg MDA/kg) at the beginning and end of storage, respectively. In the present study, the time to exceed the acceptable TBARS threshold value (1 mg MDA/kg) for consumption was more than 5 days for EnRP-treated ground beef, whereas this limit was already exceeded in the first 3 days for control samples without EnRP. These conclusions showed that lipid oxidation in aerobically packaged ground beef can be prevented by the use of EnRP. The singlet oxygen quenching, metal chelating, and free radical scavenging abilities of antioxidants such as phenolic compounds and Vit C (Table 1) in EnRP can be attributed to be responsible for the inhibition of lipid oxidation.^2,16,41^ Our results are in agreement with previous studies showing the inhibitory effect of natural antioxidants on lipid oxidation in meat product.^1,2,6^ de Souza et al.^7^ reported that ethanol and water extracts from passion fruit (Passiflora edulis) residues prevented oxidative reactions in beef burgers during storage similar to synthetic BHT. Similarly, Aksu et al.^5^ found that raspberry powder applied to vacuum-packed ground beef had a dose-dependent inhibitory effect on lipid oxidation during storage due to its high bioactive substance content. Arslan and Aksu^27^ also determined that raspberry powder exhibited strong antioxidant properties in nuggets stored at 2 ± 0.5 °C for 120 days.

### 3.6 Instrumental Color Values of Ground Beef Samples

Since the color characteristics of ground beef are an important quality criterion, minimal change during storage is desirable. The main reasons for color change in fresh ground beef ready for consumption are factors such as oxidation (myoglobin, lipid, protein), microbial activity, pH change, and drying. According to research, one of the purposes of using herbal extracts in meat products includes preventing myoglobin oxidation in processed meat products and fresh meat, thus preventing color change during storage.^8,9^ One of the aims of this study was to preserve the color values of aerobically packaged ground beef during storage. Regarding color values, L*, b*, and *h°* values of the ground beef samples were significantly (*P* < 0.01) affected by the addition of raspberry powder to ground beef, but no significant effect (*P* > 0.05) was observed on the a* and C* values. In contrast to EnRP treatment, a* (*P* < 0.01) and C* (*P* < 0.01) values were affected by storage period, and treatment x storage period interactions were found to be significant (*P* < 0.01) for both parameters (Table 3).

As shown in Table 4, the a* values of the control group and the ground beef with 1 and 2% EnRP decreased, while the values of the samples with 3% EnRP remained stable and the redness was maintained. During storage, this value representing redness decreased by 6 units in the control and 5 and 3 units in the 1 and 2% EnRP groups, respectively. On the other hand, the a* value in the 3% EnRP-added samples was determined as 20.70 at the beginning of storage and 21.56 on the fifth day of storage. Compared to initial values, 3% EnRP treatment increased redness by 4% in contrast to the redness loss of 29.13, 25.07, and 16.24% in the control, 1% EnRP, and 2% EnRP groups, respectively. These results showed that the redness value of ground beef was better preserved by the addition of EnRP during aerobic storage than by the control samples. Regarding a* value, no statistical difference (*P* > 0.05) was observed between control and EnRP-added samples on the first and third days of storage. However, on the fifth day of storage, the difference between treatments was very significant (*P* < 0.01; Table 4). As the EnRP level added to ground beef decreased, the a* value also decreased and there was a difference of approximately 6 units between the control and 3% EnRP groups. This difference was quite significant and showed that EnRP increased oxidative stability in terms of color and prevented myoglobin oxidation. In other words, the a* value was preserved, and therefore color stability was increased due to EnRP preventing myoglobin oxidation during storage. The fact that EnRP is rich in antioxidant substances and contains high levels of anthocyanins (Table 1) can be considered as responsible for both the improvement of the color properties of the ground beef and the prevention of discolouration. Anthocyanins show a pH-dependent hierarchical color change. Considering the structure of anthocyanins, the acidic pH of the EnRP is particularly important for the presentation and stability of the red color.^5,55^ In this context, lower pH values during storage in the 3% EnRP sample compared to those of the other treatment groups may have supported color stabilization. On the other hand, hydrogen bonds, hydrophobic effects, electrostatic interactions, and van der Waals forces between phenolic compounds and proteins (including myoglobin) are known to alter the structure of myoglobin.^56^ These interactions may have contributed to the longer maintenance of the bright red oxymyoglobin form by delaying the lipid oxidation that induces pigment oxidation and preventing the formation of metmyoglobin.^57^ Furthermore, anthocyanins form copigmentation with common copigment compounds (such as phenolics, amino acids, alkaloids, and organic acids) and protect the colored flavylium cation from a nucleophilic attack by water.^58^ Considering that the copigment effect is obvious under weakly acidic conditions,^58^ the copigmentation of anthocyanins in the the 3% EnRP group with other compounds may have reduced discoloration by increasing pigment stability under storage conditions. These properties of raspberry extract or powder are an important reason for the high a* value that it imparts to ground beef samples. The 3% EnRP-added group had the highest C* and lowest *h°* values at day 5 of storage (Table 4), as a result of reduced lipid oxidation and high a* values. The hue angle values of the ground beef samples increased during the storage period (*P* < 0.05). However, this increase was less in the EnRP-treated samples (Table 4). The increase in *h°* values was probably due to oxidative processes resulting in the formation of Schiff pigments from protein and lipid complexes.^6,7,59^ Similar results were reported in previous studies in which lipid oxidation and metmyoglobin formation correlated negatively with redness loss and positively with *h°* values.^5,8^

**Table 4:** Instrumental Color Values of Aerobic-Packaged Ground Beef Treated with Different Levels of Encapsulated Raspberry Powders (EnRP) during Storage at 2 ± 0.5 °C for 5 Days^a^ ^b^

		storage period (days) (SP)				
	EnRP levels	0	1	3	5	SEM
*redness* (a*)	control	22.7^aAB^	21.9^aA^	17.5^bA^	16.1^bC^	0.833
	EnRP 1%	23.9^aA^	19.7^bA^	18.2^bcA^	17.9^cBC^	
	EnRP 2%	22.4^aAB^	20.7^abA^	19.0^bA^	18.7^bB^	
	EnRP 3%	20.7^abB^	22.2^aA^	18.7^bA^	21.6^abA^	
chroma (C*)	control	27.3^aA^	25.9^aA^	23.0^bA^	21.0^bB^	0.958
	EnRP 1%	28.1^aA^	23.7^bA^	22.7^bA^	22.3^bB^	
	EnRP 2%	26.0^aAB^	23.9^abA^	23.3^bA^	22.7^bAB^	
	EnRP 3%	23.9^aB^	23.0^aA^	22.7^aA^	25.7^aA^	
hue angle (*h°*)	control	31.1^cA^	32.1^cA^	36.1^bA^	39.7^aA^	0.680
	EnRP 1%	31.5^cA^	34.6^bA^	36.6^abA^	37.4^aB^	
	EnRP 2%	30.6^bA^	34.8^aA^	35.3^aA^	34.3^aC^	
	EnRP 3%	29.9^cA^	32.4^bA^	34.7^aA^	33.3^abC^	

a Significant differences between storage periods per EnRP level are indicated by different lowercase letters in rows (*P* < .05; Duncan’s test).

b Significant differences between EnRP levels per storage period are indicated by different capital letters in columns (*P* < .05; Duncan’s test).

### 3.7 Microbial Counts of Ground Beef Samples

All microbial counts of aerobically packed ground beef samples were affected by EnRP addition (*P* < 0.01) and storage period (*P* < 0.01). Furthermore, EnRP level x storage time interactions were observed on aerobic mesophilic (*P* < 0.01), aerobic psychotrophic (*P* < 0.01), *Pseudomonas* (*P* < 0.01), *Micrococcus/Staphylococcus* (*P* < 0.05), and *Enterobacteriaceae* (*P* < 0.01) counts, except for S. aureus (*P* > 0.05) enumeration (Table 3).

The total aerobic psychotrophic, *Pseudomonas*, S. aureus, and *Enterobacteriaceae* counts were lower than other treatment groups in samples with 2 and 3% EnRP (*P* < 0.05, Table 3). The counts of total aerobic mesophilic bacteria, which were similar (*P* > 0.05) in all treatments on the first day of storage, were found to be lower (*P* < 0.05) in the samples containing EnRP on the following storage days (Table 5). The lower initial (day 0) bacterial counts in the 2% EnRP group can be attributed to the achievement of an ideal ambient pH and concentration of bioactive components, i.e., the required inhibition threshold for antimicrobial action. Similarly, lower concentrations of plant extracts have been reported to offer more effective antimicrobial activity in meat products in previous studies^1,64^ The total aerobic mesophilic bacteria count is an indicator of spoilage in meat and meat products and the maximum acceptable count is 6–7 log CFU/g.^4,7,8^ Considering that the acceptable limit for ground beef is 6 log CFU/g,^8^ total aerobic mesophilic bacteria counts in all groups containing EnRP were within acceptable limits until the end of storage (Table 5). This limit was exceeded only in the control samples on the fifth days of storage. The counts of total aerobic psychrotrophic bacteria in ground beef with EnRP were lower than in the control group after the first day of storage, and below 5 log CFU/g in ground beef with 2 and 3% EnRP. It is thought that this situation is caused by a similar change in the count of *Pseudomonas*. As seen in Table 5, the highest increase during storage was in the control samples. There was an increase in the number of control samples during storage. Although there was no significant difference between the treatments at the beginning of storage (*P* > 0.05), the *Pseudomonas* counts in the raspberry samples on the first, third, and fifth days of storage were lower than the control, which is a result of the antimicrobial effect of EnRP on *Pseudomonas*. The antimicrobial effect of EnRP on *Pseudomonas* (Table 2) was also seen in ground beef (Table 5). The change in *Pseudomonas* count was similar to the results of the previous study obtained in aerobically packed ground beef samples treated with the freeze-dried nettle water extract.^1^ Since the development of microorganisms such as *Pseudomonas*, which cause spoilage under commonly aerobic conditions, poses a significant problem in the preservation of fresh meat products, preventing their development during storage and preservation is important in terms of preserving quality. In this respect, the use of EnRP provided a significant advantage.

**Table 5:** Changes in Microbial Counts (log CFU/g) of Aerobic-Packaged Ground Beef Samples Treated with Different Levels of Encapsulated Raspberry Powders (EnRP) during Storage at 2 ± 0.5 °C for 5 Days^a^ ^b^ ^c^ ^d^

		storage period (days) (SP)				
	EnRP levels	0	1	3	5	SEM
AMB	control	4.40^cA^	4.69^cA^	5.67^bA^	6.09^aA^	0.186
	EnRP 1%	4.11^bA^	4.56^abA^	4.58^abB^	4.95^aB^	
	EnRP 2%	3.61^bB^	4.61^aA^	4.98^aAB^	4.56^aB^	
	EnRP 3%	4.13^aA^	4.52^aA^	4.51^aB^	4.42^aB^	
APB	control	4.44^cA^	4.49^cA^	5.15^bA^	5.74^aA^	0.125
	EnRP 1%	4.16^cAB^	4.48^bcA^	4.78^abAB^	5.10^aB^	
	EnRP 2%	3.89^bB^	4.40^aAB^	4.38^aBC^	4.27^aC^	
	EnRP 3%	4.37^aA^	4.14^abB^	4.15^abC^	3.98^bC^	
*Pseudomonas*	control	3.64^bA^	3.82^bA^	4.45^aA^	4.74^aA^	0.139
	EnRP 1%	3.36^bA^	3.85^bA^	3.77^bB^	4.76^aA^	
	EnRP 2%	3.16^bA^	3.40^abB^	3.83^aB^	3.78^aB^	
	EnRP 3%	3.66^aA^	3.19^bB^	3.33^bC^	3.61^aB^	
*M/S*	control	3.57^bA^	3.68^bAB^	4.78^aA^	3.74^bA^	0.158
	EnRP 1%	3.25^bA^	3.95^abA^	4.25^aAB^	3.28^bAB^	
	EnRP 2%	3.10^bA^	3.64^aAB^	3.72^aB^	3.47^abAB^	
	EnRP 3%	3.66^bA^	3.49^bB^	4.25^aAB^	3.15^cB^	
*Enterobacteriaceae*	control	2.42^bA^	2.68^bA^	3.74^aA^	4.21^aA^	0.161
	EnRP 1%	2.39^bA^	2.25^bB^	2.97^abAB^	3.50^aB^	
	EnRP 2%	2.31^bcA^	2.10^cB^	2.55^bB^	2.96^aC^	
	EnRP 3%	2.13^bcA^	2.05^cB^	2.52^aB^	2.40^abD^	

a SEM: standard error of mean.

b AMB: Aerobic mesophilic bacteria; *M/S: Micrococcus/Staphylococcus*; APB: Aerobic psychrotrophic bacteria.

c Significant differences between storage periods per EnRP level are indicated by different lowercase letters in rows (*P* < .05; Duncan’s test).

d Significant differences between EnRP levels per storage period are indicated by different capital letters in columns (*P* < .05; Duncan’s test).

EnRP prevented the growth of S. aureus (*P* < 0.01), and the highest counts were detected in control samples without EnRP (Table 3). The antimicrobial effect of EnRP on S. aureus (Table 2) was also seen in ground beef (Table 3). The effect of EnRP on S. aureus was also confirmed by Aksu et al.^5^ Staphylococcus aureus poses an important risk source in terms of contamination of various foods during production and storage. S. aureus, which can grow more easily in protein-rich foods such as fresh meat, is one of the pathogenic bacteria that is frequently seen in products prepared under unsuitable hygienic conditions, especially during the slaughter, cutting, and storage of meat.^60,61^ Many studies on meat and meat products have shown that this pathogenic bacteria is present. Guven et al.^62^ determined that 48.7% of meat products contained S. aureus. Bhargava et al.^63^ found that 65 (22.5%) of 289 raw meat samples (beef, chicken, and turkey) contained S. aureus.

As seen in Table 5, the addition of EnRP to ground beef also prevented the growth of *Enterobacteriaceae* during the storage period. Although there was no statistical difference (*P* < 0.05) between the control and with EnRP samples at the beginning of storage, lower counts were detected in EnRP samples than in control samples in the later days of storage. During storage, the difference between the 3% EnRP and control samples gradually increased, resulting in a difference of 1.81 logarithmic unit at the end of storage. These results are consistent with the previous studies.^5,10,27^ In previous studies, raspberry extracts were found to be effective against both Gram-negative and Gram-positive bacteria. Aksu et al.^5^ reported that spray-dried raspberry powder has antimicrobial effects depending on the usage level on L. monocytogenes, Micrococcus luteus, S. aureus, Klebsiella pneumoniae, Citrobacter freundii, Yersinia enterocolitica, E. coli, and P. aeruginosa. Similarly, Demirbaş et al.^50^ determined that the antimicrobial effect of raspberry water extracts was higher on Gram-negative bacteria than Gram-positive ones.

## 4 Conclusions

In this study, the effect of raspberry powder was investigated to reduce the negative effects that occur during the storage of aerobically packaged fresh ground beef at low temperatures and to maintain its quality over a longer period of time. It was determined that EnRP extends the shelf life of ground beef, thanks to its strong bioactive compound content and antimicrobial and antioxidant capacity. With the addition of EnRP to aerobically packaged ground beef, lipid oxidation and microbial spoilage were slowed and color stability was increased. EnRP showed a strong antimicrobial effect in ground beef, and the AMB count exceeded 6 log CFU/g only in control samples on the fifth day of storage. A similar change was also detected in the counts of APB, *Pseudomonas*, S. aureus and *Enterobacteriaceae*, and the counts decreased as the EnRP level increased. Lipid oxidation was prevented, depending on the amount of EnRP added to ground beef. TBARS value exceeded the 1 mg MDA/kg level in control samples between days 3–5 and the 2 mg level on day 5. This value decreased depending on the EnRP addition level in the samples with EnRP addition and did not exceed the acceptable limit during storage for 5 days of storage. No significant differences were observed between 2% and 3% EnRP levels in many parameters examined (microbiology and TBARS). Overall results showed that EnRP can contribute to the shelf life extension of aerobically packed ground beef. Considering the antimicrobial, antioxidant, pH and color properties of EnRP, the inclusion of 2% EnRP can be recommended for the preservation of fresh ground beef. Furthermore, future studies are needed to evaluate the effects of EnRP on fresh meat products under different packaging (modified atmosphere, smart packaging, etc.,) conditions and over longer storage time.
